# Updates and future directions regarding hand hygiene in the healthcare setting: insights from the 3rd ICPIC alcohol-based handrub (ABHR) task force

**DOI:** 10.1186/s13756-024-01374-9

**Published:** 2024-02-29

**Authors:** Ermira Tartari, Fernando Bellissimo-Rodrigues, Daniela Pires, Carolina Fankhauser, Nasim Lotfinejad, Hiroki Saito, Miranda Suchomel, Axel Kramer, Benedetta Allegranzi, John Boyce, Hugo Sax, Andrew J. Stewardson, Didier Pittet, the ICPIC Alcohol-Based Handrub Task Force, Aeschbach Rodin, Kumashita Yuichi, Alléard Benjamin, Lee Pamela, Lu Tingxu, Arbogast Jim, Mermel Leonard, Azzouz Chedly, Barrett Emma, Park Ben, Quan Lelyn, Bühler Martina, Capilna Andreea, Serna Jiménez César, Damani Nizam, Eggerstedt Sven, Teska Peter, van Hall Nicole, Hansen Sonja, Vos Margreet, Hennig Thomas, Widmer Andreas, Herwaldt Loreen, Yakata Kazuko, Yokoe Deborah, Bell Mike, Bertho Jean Noel, Borzykowski Tcheun-How, Conly John, Da Silva Carlos, Yakata Kazuko, Geva Ariel, Haidegger Tamas, Herwaldt Loreen, Holland Carolyn, Okeke Bonnie, Ormandy Kevin, Parneix Pierre, Peters Alexandra, Pouget Vanessa, Robert Lionel, Serna Jimenez Cesar, Zingg Walter

**Affiliations:** 1 Faculty of Health Sciences, University of Malta, Msida, Malta; 2 Infection Prevention and Control Technical and Clinical Hub, Department of Integrated Health Services, World Health Organization (WHO), Geneva, Switzerland; 3 Social Medicine Department. Ribeirão Preto Medical School, University of São Paulo, Ribeirão Preto, Brazil; 4 National Institute of Health and Care Research, Health Protection Research Unit in Healthcare Associated Infections and Antimicrobial Resistance, Imperial College London, London, UK; 5 Clean Hospitals, Geneva, Switzerland; 6 Infection Control Program and WHO Collaborating Centre, Faculty of Medicine, Geneva University Hospitals, Geneva, Switzerland; 7 Department of Emergency and Critical Care Medicine. Faculty of Medicine, Institute of Global Health, . Mariana University Yokohama Seibu Hospital, University of Geneva, Geneva, Switzerland; 8 Institute of Hygiene and Applied Immunology, Medical University of Vienna, Vienna, Austria; 9 Institute of Hygiene and Environmental Medicine, University Medicine Greifswald, Greifswald, Germany; 10 J.M. Boyce Consulting, LLC, Hyde Park, NY USA; 11 Department of Infectious Diseases, Bern University Hospital and University of Bern, Bern, Switzerland; 12 Department of Infectious Diseases, Central Clinical School, The Alfred Hospital, Monash University, Melbourne, Australia; 13 Faculty of Medicine & Clean Hospitals, University of Geneva, Geneva, Switzerland

**Keywords:** Infection prevention and control, Hand hygiene, Alcohol-based handrub, Healthcare-associated infections, Antimicrobial resistance, Technique, Volume, Microbial load, Skin microbiome

## Abstract

Healthcare-associated infections (HAIs) and antimicrobial resistance (AMR) pose threats to global health. Effective hand hygiene is essential for preventing HAIs and the spread of AMR in healthcare. We aimed to highlight the recent progress and future directions in hand hygiene and alcohol-based handrub (ABHR) use in the healthcare setting. In September 2023, 42 experts in infection prevention and control (IPC) convened at the 3rd International Conference on Prevention and Infection Control (ICPIC) ABHR Taskforce in Geneva, Switzerland. The purpose of this meeting was to provide a synthesis of recent evidence and formulate a research agenda on four critical areas for the implementation of effective hand hygiene practices: (1) ABHR formulations and hand rubbing techniques, (2) low-resource settings and local production of ABHR, (3) hand hygiene monitoring and technological innovations, and (4) hand hygiene standards and guidelines.

## Background

### Alcohol-based handrub (ABHR) task force

The International Conference on Prevention and Infection Control (ICPIC) hand hygiene alcohol-based hand rub (ABHR) Task Force represents a critical platform that builds upon the World Health Organization (WHO) Guidelines on Hand Hygiene in Healthcare [1]. Established in 2017, the ICPIC ABHR Task Force aims to review new knowledge and discuss gaps and future research priorities. This meeting was held on 15 September 2023 immediately following the end of ICPIC in Geneva, Switzerland. ICPIC is a well established entity in the field of international infection prevention and control (IPC), attracting participants from over 100 countries worldwide since 2011*,* offering a unique forum for the exchange of knowledge and expertise in the prevention of healthcare-associated infections (HAIs) and the control of antimicrobial resistance (AMR), patient safety, and global health.

The ABHR Task Force working group has significantly influenced policy and practice by successfully lobbying for the reclassification of ethanol, a critical component of handrubs [2]. This accomplishment has not only facilitated wider access to essential hand hygiene products but has also underscored the importance of evidence-based advocacy in public health initiatives. Moreover, the ABHR Task Force working group has recently played a crucial role in establishing International Organization for Standardization (ISO) standards for hand hygiene in healthcare facilities [3]. Their work is instrumental in advancing the quality and effectiveness of IPC strategies globally.

### 3rd ICPIC task force meeting

Building on the successful discussions of the previous two ICPIC ABHR Task Force meetings held in 2017 and 2019 and recognizing the challenges posed by SARS-CoV-2, the 3rd ICPIC Hand Hygiene ABHR Task Force meeting addressed four key domains: (1) alcohol-based handrub formulations and handrubbing technique; (2) hand hygiene monitoring technology and promotion; (3) low-resource settings and local production of alcohol-based handrub; and (4) hand hygiene standards and guidelines. The selection of participants was based on their expertise and reputation in the field of IPC and hand hygiene, identified through literature searches and Geneva University Hospitals stakeholder networks. The meeting was structured around focused topics, including presentations on the latest evidence, followed by updates on research from a diverse range of stakeholders, including academia, industry, and IPC experts. Following the presentations in each domain, the experts were given time for discussion, which was moderated, and notes were taken. The goal was to obtain a comprehensive inventory of the current state of the art, highlighting areas where further research is needed. The experts were invited to express their opinions and concerns, and to identify gaps in the literature.

## Alcohol-based handrub formulations and hand rubbing technique

### Efficacy and toxicological features

ABHR kills the entire spectrum of vegetative bacteria, yeasts, and molds but not bacterial spores or parasites. Ethanol-based handrubs (EBHR) have strong efficacy in killing non-enveloped viruses, whereas 1-propanol- and 2-propanol-based handrubs do not kill non-enveloped viruses. SARS-CoV-2, an enveloped virus, has shown high susceptibility to ethanol, 1-propanol, and 2-propanol [4–7]. Hand hygiene using ABHR is thought to contribute to the prevention of SARS-CoV-2 transmission both in the community and in the healthcare setting [8]. However, COVID-19 transmission results from both direct contact and respiratory transmission and the relative importance of each mode of transmission remains to be clarified in well-designed and appropriately conducted research.

The amount of ethanol absorbed through the skin during hand hygiene is similar to the consumption of beverages with hidden ethanol content (< 0.5% v/v), such as apple juice or kefir. There is no risk of carcinogenicity, mutagenicity, or reproductive toxicity due to the repeated use of EBHR. The amount of absorbed propanoles is low, and no evidence of toxicity exists [2].

**Table:** 

Identified gaps	
The precise minimum inoculum necessary for skin-to-skin transmission has not been determined. Further research in this area is essential to optimize hand hygiene techniques, volume, and duration.	

### Redefining hand hygiene action ("how to handrub"): technique, volume, and duration

Currently, there is no established consensus in the literature regarding the ideal technique, volume, or duration of hand hygiene. Ideally, an optimal hand hygiene action would prevent the transmission of pathogens through the hands of healthcare workers. However, defining this concept is challenging because of varying levels of hand contamination among healthcare workers and the minimum inoculum required for transmission. The level of hand contamination among healthcare workers depends on clinical activity, and the minimum inoculum for transmission may vary among different pathogens and bacteria. A study conducted in a laboratory setting using *Escherichia coli (E. coli)* demonstrated that only a 1 log_10_ minimum inoculum was required for transmission. As hand contamination in clinical care rarely exceeds 3 log_10_, it is hypothesized that a 2 log_10_ reduction, achieved through hand hygiene, is sufficient to prevent transmission [9].

### Hand hygiene technique

To date, efforts aimed at improving hand hygiene have primarily targeted increasing compliance rates with hand hygiene opportunities. However, the quality and technique of hand hygiene action, or the “How to Handrub” method, which is essential to assure complete coverage of hands with ABHR, has been overlooked [10–12]. The scarcity of studies evaluating hand hygiene techniques in clinical settings coupled with the absence of a standardized monitoring tool for assessing adherence to the technique, highlights a gap in current hand hygiene practices. An observational study at the University Hospital Basel reported a stark contrast between hand hygiene performed in 93.2% of all opportunities and markedly low adherence to the WHO 6-step hand hygiene technique at only 8.5% [10]. This indicates a critical need for further studies to assess hand hygiene techniques in clinical settings and guide healthcare workers' attention to the most contaminated hand parts.

**Fig. 1:**
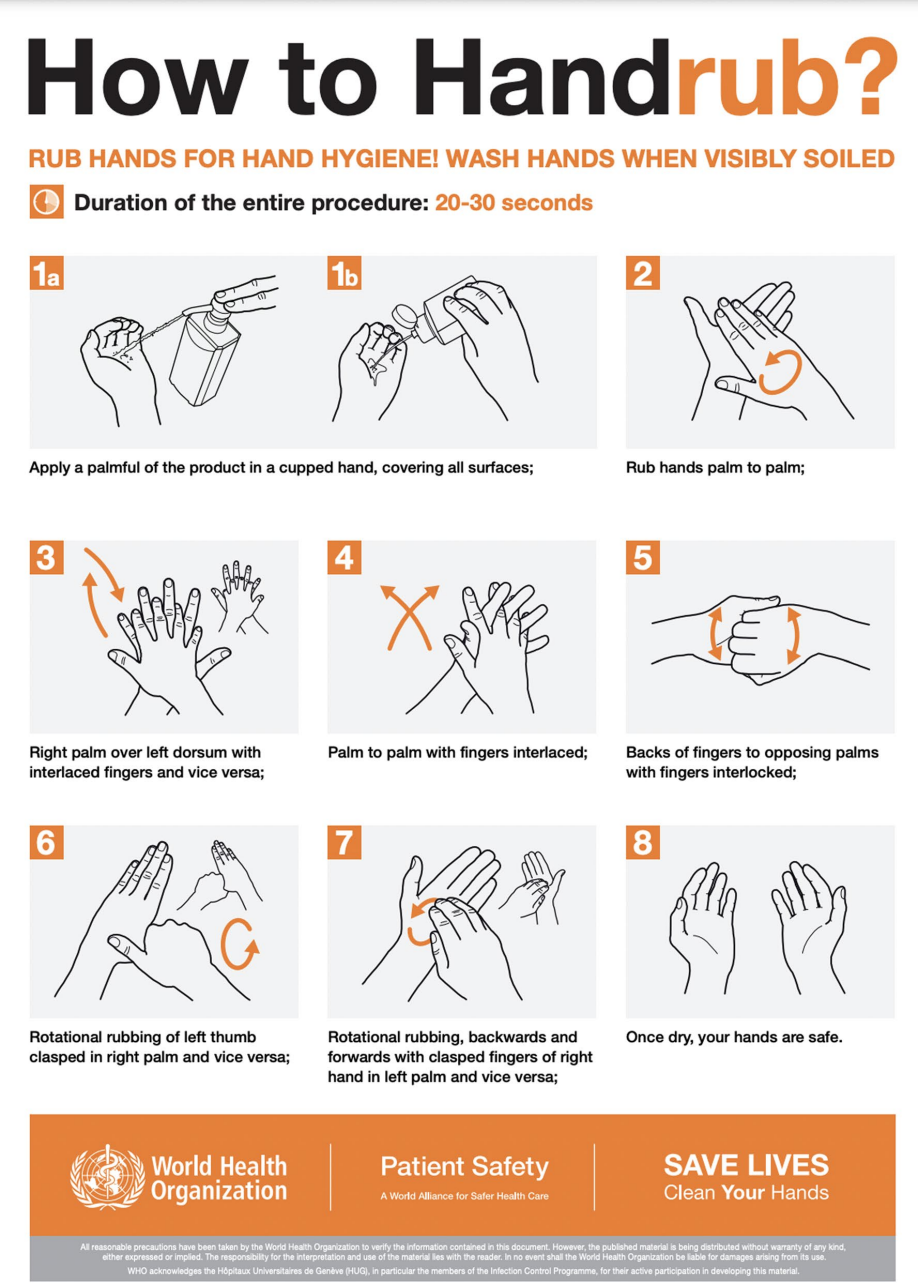
World Health Organization’s 6-step technique for hand rubbing with alcohol-based handrub

**Fig. 2:**
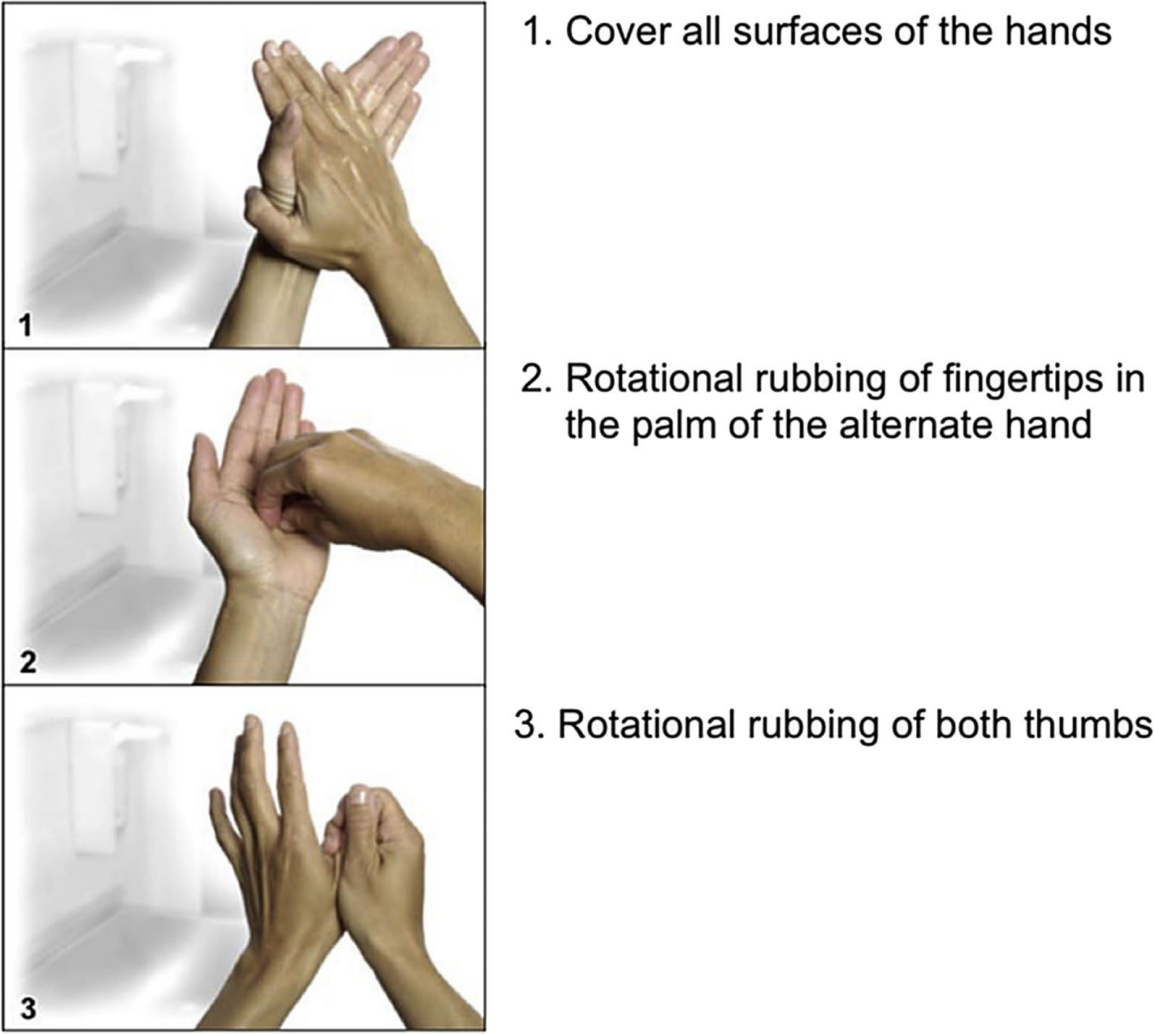
Simplified 3-step hand hygiene technique. (From Tschudin-Sutter S, Rotter ML, Frei R, Nogarth D, Häusermann P, Stranden A, Pittet D, Widmer AF. Simplifying the WHO ‘how to hand rub’technique: three steps are as effective as six—results from an experimental randomized crossover trial. Clinical Microbiology and Infection. 2017 Jun 1;23(6):409-e1; with permission)

Two hand hygiene techniques, the "6 steps" method recommended by the 2009 WHO Hand Hygiene Guidelines (Fig. 1) [1] and the “3 steps” technique originally proposed by the CDC [13] and subsequently adapted by the University Hospital Basel, Switzerland (Fig. 2) [14], have been studied in recent years. Both techniques have been shown to be equally effective in reducing the microbial load on the hands, but the “3 steps” technique is easier to remember and promote [12, 14, 15]. However, a systematic review conducted by Price et al. [16] found insufficient evidence to determine the most effective and feasible hand hygiene technique in a real clinical setting, and called for further robust research on the reproducibility of these findings in clinical settings.

**Table:** 

Identified gaps	
Compliance with the hand hygiene technique or “How to Handrub” has not been adequately evaluated.	
Observational studies to assess hand hygiene technique in clinical settings are needed.	
There is inconclusive evidence regarding the most effective hand hygiene technique in “real world” clinical settings. Effectiveness in this context incorporates elements of both microbiological efficacy and implementation.	

### Alcohol-based handrub (ABHR) volume

The antimicrobial efficacy of ABHR is affected by several factors other than the technique used, such as the volume of product applied, duration of handrubbing (drying time), and hand size. Healthcare workers frequently apply suboptimal amounts of ABHR and use a poor hand hygiene technique that may not be sufficient to cover all surfaces of the hands and achieve desired bacterial load reduction [10, 17–20]. WHO guidelines recommend that “a palmful” of the product is sufficient to cover all surfaces” while the CDC recommends using the manufacturer-recommended volume. Current studies suggest that the greater the volume of ABHR applied, the higher is the antimicrobial efficacy [21–23]. While the ABHR volume recommended by the European Norm 1500 [24] and most manufacturers is of 3 mL, there is concern that the mean application volume of ABHR in the clinical settings is lower than 1 mL [18–20, 25]. A study reported that more than 86% of Dutch and Canadian healthcare workers used a single pump of ABHR product, whether it is set at 0.75 mL or 1.5 mL, suggesting ABHR dispensers should be adjusted to provide the recommended volume of ABHR in one single pump [19]. ABHR volumes of less than 1 mL often result in dry time of less than 15 s, while studies suggest that dry times of 15 s or longer should be applied to achieve bacterial load reduction [23].

Smart technologies in the future could provide the ideal ABHR volume that is linked to the hand size and adheres to “one size does not fit all”, ABHR volume should be customized to each health worker’s hand surface size [2, 23, 26]. Future studies are needed to understand application volumes used by clinicians in practice and to assess the efficacy of ABHR at such volumes since the majority of studies are lab-based research.

**Table:** 

Identified gaps	
Determine the ABHR volume required to prevent infection transmission in clinical settings.	
How can ABHR volumes be customized to achieve adequate antimicrobial efficacy and dry times for individuals with different hand sizes?	

### ABHR application time (duration of friction)

Recent studies suggest that the optimal duration of handrubbing with ABHR to ensure adequate hand decontamination is at least 15 s [27, 28]. With the recommended technique, the palms are covered in 15 s in the same manner as with 30 s of rubbing [29]. Studies involving volunteers following experimental contamination with *E. coli* [12, 13] and in the daily routine of a neonatology and gynaecology ward have shown non-inferiority of 15 s rubbing time compared to the standard of 30-s for application of ABHR. Reducing the application time is associated with increased frequency of ABHR usage and hand hygiene compliance [19, 30, 31] and would be practical and time saving for healthcare workers. However, when observing adherence to hand hygiene technique, only 7% of healthcare workers attained full coverage of all hand surfaces, with the thumb and fingertips being the most commonly missed areas [32]. Therefore, when changing from 30 to 15 s, the attainment of full hand surface coverage should be included in the evaluation of hand hygiene quality [33].

The volume of ABHR applied and the duration of friction affect the dry time (how long the hands must be rubbed before they feel dry), which, in turn, affects the antimicrobial efficacy of the handrub [10]. A systematic review [34] found insufficient evidence to change current hand hygiene guideline recommendations regarding ABHR volume and application time.

**Table:** 

Identified gaps	
To establish the optimal duration for handrubbing and to develop ABHR formulations with a faster drying time, in order to prevent healthcare workers from using insufficient amounts of ABHR that dry quicker.	
To examine the relationship between the ABHR volume applied and the duration of friction in order to determine the optimal duration for handrubbing and ideal formulation for effective hand hygiene practices.	

### Liquid, gel, foam: evidence of their effectiveness in preventing infections

There is considerable discourse regarding the effectiveness of ABHR gels or foams in reducing HAIs. Of note, the 2009 WHO Guidelines for Hand Hygiene in Health Care and the 2022 SHEA/IDSA/APIC Practice Recommendation regarding hand hygiene strategies both include liquid, gel, or foam as options for the selection of ABHRs. Several factors affect the antimicrobial efficacy of ABHR, including the volume of ABHR applied to the hands, dry time, ABHR formulation, and laboratory method(s) used to evaluate antimicrobial efficacy [21–23, 35]. However, the format of currently available ABHR products does not have a major impact on antimicrobial efficacy. Data on the impact of ABHR format on dry time is not consistent. Two studies found that the ABHR format had no significant impact on dry times [22, 36]. Other studies reported that dry times were shorter with rinses [37] or gels [38], and one study found that dry times were not significantly different for liquids and foams but were longer with gels [27]. Multiple other factors are likely to affect ABHR effectiveness in reducing HAIs and transmission of healthcare pathogens [39, 40]. Unfortunately, no prospective controlled trial has compared the effectiveness of different ABHR formats in reducing HAIs. A review of 41 studies revealed that all ABHR formats have been successful in improving hand hygiene compliance rates when combined with multimodal improvement strategies, with varying abilities to yield significant reductions in HAIs or pathogen transmission (manuscript in preparation). However, differences in the frequency of significant HAI reduction achieved by liquid, gel and foam formats were not statistically significant.

**Table:** 

Identified gaps	
The absence of sufficient “in-vivo” laboratory studies to evaluate the antimicrobial efficacy of ABHRs.	
Clinical studies including healthcare providers involved in direct patient care are essential to evaluate the impact of skin tolerability, acceptability, application frequency and compliance on HAIs.	
The lack of randomized controlled trials to determine the effectiveness of various ABHR formats in reducing HAIs and pathogen transmission.	

### Efficacy testing of ABHRs in the lab: advantages and caveats

It is necessary to evaluate the antimicrobial efficacy of ABHRs before it can be used in ‘real-life’ clinical settings [1, 13]. However, the efficacy of ABHR can also depend on the individual using it, which is why *in-vivo* efficacy tests under simulated practice conditions on artificially contaminated hands of volunteers under simulated practice conditions are required.

The European in vivo test model EN 1500 [41] utilizes an internal reference treatment on the same volunteers as their own control, which meets the statistical requirements of smaller sample sizes than the American ASTM E2755 test model [42]. In contrast to EN 1500, in which hands are contaminated by immersion in a bacterial suspension, resulting in significant skin contamination, ASTM E2755 uses a "low volume" contamination method. The reduced volume leaves the hands dry and minimally soiled when the ABHR is applied, allowing typical product volumes to be tested at more realistic product drying times. Both in-vivo test models use test organisms for contamination – *E coli* (EN 1500) and *Serratia marcescens* (ASTM E2755)—which are not considered the best representatives of hand-transmitted pathogens.

Critics argue not only that the type of contamination according to EN 1500 is problematic, but also that the test organisms of the two standards (EN and ASTM) are not appropriate. Moreover, the choice of the reference treatment EN 1500, which is 60% (v/v) propan-2-ol (2 × 3 mL/2 × 30 s), has been questioned because the majority of ABHR is applied once only and for a maximum of 30 s [43], which is more consistent with daily practice in healthcare.

**Table:** 

Identified gaps	
The development of improved test methodologies to evaluate the *in-vivo* efficacy of ABHR that accurately reflects clinical use, and the use of contamination techniques that align with hand contamination patterns in clinical settings.	
Selection of test organisms with high environmental stability and hand-transmissibility.	
Laboratory studies utilizing adapted test methodologies, to demonstrate sufficient *in-vivo *efficacy of ABHR.	

Despite many lab-based studies, research is scarce evaluating the efficacy of ABHRs in reducing organisms acquired on hands in real-world settings using real-world techniques.

### Skin microbiome and ABHRs

The hand microbiome is a complex ecosystem inhabited by bacteria, archaea, fungi, other microbial eukaryotes, and viruses, comprising less than 20 proportion of viruses and fungi [44].

The skin microbiome displays interpersonal, gender-specific and time-dependent variances [43]. Because the influence of daily ABHR on skin microbiome is unknown, the short-term effect of EBHR was determined in a prospective clinical trial. After paid leave for 14 days without hand antisepsis, samples were collected on the first working day before the first handrub was used, at the end of the shift, and on days 7 and 28 at the beginning and the end of shift. On average, hand antisepsis was performed 112 times per work shift. The microorganisms were collected using the glove-juice technique. The pro- and eukaryotic community profiles were created using amplicon sequencing of 16S and 18S rRNA markers. Among the prokaryota, 2667 phylotypes with 587 genera and among eukaryotas, 427 species with 118 genera were identified. For prokaryota, daily exposure led to the end-of-the-day microbiomes being more similar to each other across nurses. In contrast, the longitudinal effect of the 28-day application revealed greater similarity in the eukaryotic community. Frequent use of EBHR has no detrimental effects on the hand microbiome [45].

**Table:** 

Identified gaps	
To evaluate the impact of isopropanol- or n-propanol based ABHR on the skin microbiome, as well as the consequences of incorporating long-lasting antimicrobial agents, such as chlorhexidine, into ABHR formulations.	
Ideally, both culture-dependent and and culture-independent methods should be used to assess the hand microbiome, as each method has advantages and disadvantages.	

## ABHR accessibility: low-resource settings and local production

### Building local ABHR production and promotion in low-resource countries

The promotion and production of ABHR in low-resource countries were examined. Currently, there is a scarcity of studies describing local ABHR production in low-resource settings. A study conducted by the WHO in 2013 provided valuable insights into this area; however, an update is required [46].

Two types of local ABHR production were discussed: facility-level production, which involves trained staff such as pharmacists producing ABHR at their own facilities, and locally-manufactured ABHR, which is produced in a factory using locally sourced materials such as sugarcane and maize. An example of the latter was provided by Uganda, where a hygienic company manufactures ABHR in a factory in collaboration with a local sugar factory and distributes it to health facilities in the country as well as some neighboring countries. A cluster-randomised trial is currently underway to evaluate the effect of locally manufactured ABHR on hand hygiene promotion in the Eastern part of Uganda. Both approaches are crucial for improving access to ABHR at facility level and for implementing the WHO's multimodal hand hygiene improvement strategy. It is essential that further actions are taken to improve hand hygiene in healthcare in low-resource settings [27].

**Table:** 

Identified gaps	
To overcome challenges in local ABHR production and quality assurance.	
To develop sustainable strategies for ABHR production during crises.	

## Hand hygiene monitoring and technological innovations

### Direct observation method and the Hawthorne effect

Direct hand hygiene observations conducted by trained observers are widely regarded as the “gold standard” for estimating hand hygiene compliance. This method differs from others in that it enables the monitoring of all “Five Moments for Hand Hygiene” [47]. Assessing the quality of direct observation is crucial given the effort, time, and personnel involved. The Hawthorne effect, which occurs when individuals modify their behavior because of their awareness of being observed, is considered a significant bias [48]. Hence, standardized methodologies are crucial for measuring its extent in diverse care and clinical settings and for identifying effective interventions that can minimize it [48–50].

The Hawthorne effect is influenced by three factors: a high number of opportunities for observation during healthcare provision, low baseline compliance with hand hygiene, and ease of compliance [49]. Methods such as limiting observation periods, habituation, comparing compliance between covert and overt observations, and comparing automated hand hygiene monitoring systems (AHHMS) with direct observation can be used [51–53]. Some studies have suggested that the AHHMS can overcome this bias. A recent study reported a high level of precision between direct observation and AHHMS, whereas direct observation with hand hygiene adherence was threefold higher [53]. Non-published data from a study where healthcare workers were observed frequently for six months indicate that habituation may occur after the third observation.

**Table:** 

Identified gaps	
To establish methods to improve the accuracy of direct observations.	
To establish methods that assess and allow for the Hawthorne effect to be quantified.	
To evaluate differences in Hawthorne effect between clinical specialties.	

### Automated hand hygiene monitoring systems (AHHMSs)

The standard of care for monitoring hand hygiene compliance and quality remains direct observation. AHHMSs have been suggested as a supplement to direct observations. However, the accuracy of these automated systems has not been well-documented in the literature [54]. In a recent systematic review and meta-analysis conducted by Geneva University Hospitals, only 10% of the relevant studies provided data on the accuracy of electronic monitoring systems in detecting hand hygiene compliance or quality (unpublished data). Most studies used real-time locating systems to monitor hand hygiene compliance. Few studies have provided information on the algorithms used to detect hand hygiene compliance/quality. This study demonstrated the high sensitivity and specificity of AHHMSs. Most studies monitored adherence to Moments 1, 4, and 5 of the Five Moments for Hand Hygiene [47]. In studies that considered two or three moments, the systems were often unable to differentiate between the studied moments (e.g. between moments 3 and 4 or moments 4 and 5) [53, 55, 56]. Currently, there is no system designed to assess the colonization risk in a hospitalized patient. Such a system would need to consider all hand hygiene actions before patient contact, not just the last action between the healthcare zone and the patient.

Recently, a monitoring system was implemented, which included a portable transponder designed to detect the use of ABHR to provide feedback, a beacon recognizing entries to and exits from the patient's surroundings, and a sensor placed at the hand-rub dispensers to count the number of hand rubs. Moments 1, 4, and 5 were also identified. The system was used in a cross-over design with a 6-week each intervention, no intervention, or intervention. An increase in adherence of up to 104.5% was observed. Upon cessation of the intervention, adherence levels decreased to less than or equal to the baseline measurement. Thus, short-term intervention alone is insufficient to lead to long-term changes in hand hygiene adherence. Rather, permanent feedback and/or integration into a multimodal intervention strategy is necessary [57]. The integration of AHHMSs into a multimodal hand hygiene improvement strategy is of paramount importance. While these systems can provide many more hand hygiene events than direct observations, it is essential to recognize that their deployment alone may not result in substantial or enduring improvement in hand hygiene practices.

**Table:** 

Identified gaps	
Evaluate the accuracy of automated hand hygiene monitoring systems in multiple healthcare settings, according to the WHO “5 moments” concept.	
Develop automated systems capable to accurately assess the quality of hand hygiene action.	
Provision of methodological protocols for research evaluating the accuracy of automated systems.	
Guidance on the definition of true and false hand hygiene events and the statistical measures appropriate for evaluating accuracy.	
Guidance on the classification of automated systems (existing classification methods vary).	
Develop and validate a system to evaluate the colonization risk in hospitalized patients considering all hand hygiene actions.	
Create a model to predict the colonization risk associated with patient interactions.	

### Patient hand hygiene

Although the majority of initiatives aimed at improving hand hygiene in healthcare facilities have primarily targeted healthcare workers, there has been growing awareness of the importance of hand hygiene among patients and visitors since the emergence of COVID-19. Patients who are colonized or infected with pathogenic microorganisms can contribute to cross-transmission of these organisms to surfaces or the hands of healthcare workers [58, 59]. Various interventions such as implementing a multimodal strategy for hand hygiene improvement or involving nursing home residents as hand hygiene champions for other residents, have demonstrated effectiveness in improving patient hand hygiene [60–62]. Improving patient hand hygiene has been shown to reduce respiratory virus outbreaks, methicillin-resistant *Staphylococcus aureus* infections, vancomycin-resistant enterococcus multicentre outbreaks, and *Clostridioides difficile* infections, despite the fact that ABHR has no effectiveness on *C. difficile* [63–66]. In a randomised control trial, patients randomised to a 4-moment hand hygiene intervention were significantly less likely to acquire healthcare-associated pathogens on their hands than those receiving standard care (2% vs. 34%) [62]. However, a pilot randomized trial found that a 5-moments for patient hand hygiene did not significantly reduce the acquisition of colonization with pathogenic microorganisms [67].

While embracing patients as active stakeholders in hand hygiene improvement strategies may be beneficial, further studies are required to establish the impact of such interventions.

**Table:** 

Identified gaps	
To evaluate the impact of patient hand hygiene interventions on colonization or infection with pathogenic microorganisms.	

## Hand hygiene education and training through serious games

Several WHO surveys identified “training and education” to support hand hygiene and IPC practices improvement as one of the least implemented core component of IPC programmes or element of the hand hygiene improvement strategies [68], in particular in low- and middle-income countries. Therefore, WHO strongly encourages countries to prioritize scaling-up training and education using innovative approaches to stimulate participation and help knowledge acquisition [69]. Serious game technologies are increasingly used in healthcare as educational applications for teaching, learning, communication, or even information spreading. With this goal, the WHO developed and launched the *"My Five Moments – The Game",* a forward-thinking serious game designed to train healthcare professionals in hand hygiene practices, aligning with WHO's Five Moments for Hand Hygiene [47]. This game is set within an International Alien Hospital located in a futuristic world 200 years ahead of our time. It immerses players in lifelike scenarios where they are tasked with caring for alien patients and executing hand hygiene at pivotal moments according to the 'My Five Moments for Hand Hygiene' concept while demonstrating professional compassion and navigating the healthcare and patient zones and critical sites. This game balances educational objectives with engaging aspects of gaming, thereby contributing to growing initiatives aimed at enhancing IPC knowledge in an appealing manner.

The game's initial three levels were launched during the World AMR Awareness Week, November 18–24, 2023. The final two levels are scheduled for release on the World Hand Hygiene Day in May 2024. The development team invites players to experience the game and share feedback through the provided link [70].

**Table:** 

Identified gaps	
A significant yet unexplored aspect is the extent to which experiences from the game translate into real-world practice. Consequently, there is a need for well-structured, randomized comparative studies to evaluate the impact of such hand hygiene-focused serious games on the actual hand hygiene behaviors of healthcare providers.	

## Hand hygiene standards and guidelines

### What’s new in the 2022 SHEA guidelines on hand hygiene

The 2022 SHEA/IDSA/APIC Practice Recommendation [33] on hand hygiene strategies includes new elements regarding the following topics: selection of appropriate products, ensuring accessibility of hand hygiene supplies, use of multiple methods of monitoring compliance and providing feedback, maintenance of healthy skin and fingernails, appropriate glove use, methods for reducing environmental contamination associated with sinks and sink drains, and approaches that should not be considered a routine part of hand hygiene.

### Driving the international hand hygiene research agenda for 2023 and beyond

WHO has released a summary of the research agenda for hand hygiene in healthcare for the period 2023–2030 [71] and intends to publish a more detailed account of its contents in the near future. The goal of the agenda is to accelerate knowledge generation regarding the best interventions to improve hand hygiene practices, which will improve the quality of care and patient outcomes and reduce the risk of HAIs and AMR. The research agenda provides guidance to researchers, policy-makers, and donors by focusing on six core hand hygiene domains: system change, training and education, evaluation and feedback, reminders and communication, institutional safety climate, and the impact of hand hygiene on HAIs/AMR. The highest research priorities include identifying approaches or interventions needed to facilitate sustained system change; assessing the efficacy of hand hygiene agents in removing a range of organisms; evaluating the impact of different hand hygiene training and educational strategies; assessing the use of data feedback on barriers to and predictors of hand hygiene compliance; and determining the association between an increase in hand hygiene compliance and a reduction in transmission, colonisation, and/or infection by microorganisms of interest. The research agenda is a useful tool for researchers and donors to direct their investments in areas of hand hygiene research that still have significant gaps. Ultimately, this agenda will contribute to improving hand hygiene compliance and ensuring quality patient care [71].

### ISO23447:2023

The International Organization for Standardization (ISO) aims to develop globally recognized standards to advance the implementation of innovation across industries. In June 2018, the ISO/TC 304 Working Group (WG) 3—Infection Prevention Management was established to set standards for hand hygiene training, compliance benchmarking, performance and feedback, and requirements for healthcare facilities. These new standards aim to provide normative references, establish a standard process for hand hygiene training and assessment, clarify terminology and definitions, offer a sound rationale for hand hygiene, and integrate existing scientific knowledge into comprehensive evidence-based guidelines. These standards answer three key questions regarding hand hygiene: When, how, and what to perform. WG 3 completed its work in May 2023, holding 72 meetings from January 2018 to April 2023 (monthly before the pandemic, bi-weekly during the pandemic, and weekly thereafter). ISO/TC304 AWI 23447 was approved by TC 304 in September 2023 and subsequently published as an international standard in December 2023 [3].

## Conclusion

The third meeting of the ICPIC ABHR Task Force played an important role in furthering our understanding of hand hygiene. With a focus on critical domains such as ABHR formulations, handrubbing techniques, and a serious game for hand hygiene, the meeting highlighted both achievements and existing gaps. The identified research gaps underscore the pressing need for additional research, ranging from understanding the skin microbiome to determining the optimal hand hygiene technique. The efficacy of different ABHR formats and their impact on HAIs deserve further attention.

Addressing challenges in low-resource settings and local ABHR production emphasized the importance of sustainable strategies during crises. Moreover, updated guidelines, the release of the WHO research agenda, and new ISO norms for hand hygiene signify a commitment to global efforts to enhance infection prevention practices, reduce infections, and combat AMR. The collective knowledge shared at this task force meeting serves as a catalyst for continuous collaboration and advancement in IPC.

## Data Availability

Not applicable.

## Abbreviations

AMR: Antimicrobial resistance
HA: Healthcare-associated infection
IPC: Infection prevention and control
WHO: World Health Organization

## Declarations

### Disclaimer

The authors are responsible for the views expressed in this article, and they do not necessarily represent the views, decisions, or policies of the institutions with which they are affiliated. D Pires: The author is affiliated with the National Institute for Health Research Health Protection Research Unit (NIHR HPRU) in Healthcare Associated Infections and Antimicrobial Resistance at Imperial College London in partnership with the UK Health Security Agency (previously PHE) in collaboration with, Imperial Healthcare Partners, University of Cambridge and University of Warwick. The views expressed in this publication are those of the author(s) and not necessarily those of the NHS, the National Institute for Health Research, the Department of Health and Social Care or the UK Health Security Agency.

## References

[1] World Health Organization (2009). WHO guidelines on hand hygiene in health care: first global patient safety challenge clean care is safer care.

[2] Kramer A, Arvand M, Christiansen B, Dancer S, Eggers M, Exner M, Müller D, Mutters NT, Schwebke I (2022). Ethanol is indispensable for virucidal hand antisepsis: memorandum from the alcohol-based hand rub (ABHR) Task Force, WHO Collaborating Centre on Patient Safety, and the Commission for Hospital Hygiene and Infection Prevention (KRINKO), Robert Koch Institute, Berlin, Germany. Antimicrob Resist Infect Control.

[3] ISO 23447:2023. Healthcare organization management; Hand hygiene performance. 2023. [Cited:15 December]. https://www.iso.org/standard/75612.html?browse=tc.

[4] Leslie RA, Zhou SS, Macinga DR (2021). Inactivation of SARS-CoV-2 by commercially available alcohol-based hand sanitizers. Am J Infect Control.

[5] Golin AP, Choi D, Ghahary A (2020). Hand sanitizers: a review of ingredients, mechanisms of action, modes of delivery, and efficacy against coronaviruses. Am J Infect Control.

[6] Kratzel A, Todt D, V'Kovski P, Steiner S, Gultom M, Thao TTN, Ebert N, Holwerda M, Steinmann J (2020). Inactivation of severe acute respiratory syndrome coronavirus 2 by WHO-recommended hand rub formulations and alcohols. Emerg Infect Dis.

[7] Singh D, Joshi K, Samuel A, Patra J, Mahindroo N (2020). Alcohol-based hand sanitisers as first line of defence against SARS-CoV-2: a review of biology, chemistry and formulations. Epidemiol Infect.

[8] Hirose R, Ikegaya H, Naito Y, Watanabe N, Yoshida T, Bandou R, Daidoji T, Itoh Y, Nakaya T (2021). Survival of severe acute respiratory syndrome coronavirus 2 (SARS-CoV-2) and influenza virus on human skin: importance of hand hygiene in coronavirus disease 2019 (COVID-19). Clin Infect Dis.

[9] Bellissimo-Rodrigues F, Pires D, Soule H, Gayet-Ageron A, Pittet D (2017). Assessing the likelihood of hand-to-hand cross-transmission of bacteria: an experimental study. Infect Control Hosp Epidemiol.

[10] Tschudin-Sutter S, Sepulcri D, Dangel M, Schuhmacher H, Widmer AF (2015). Compliance with the World Health Organization hand hygiene technique: a prospective observational study. Infect Control Hosp Epidemiol.

[11] Stewardson AJ, Iten A, Camus V, Gayet-Ageron A, Caulfield D, Lacey G, Pittet D (2014). Efficacy of a new educational tool to improve Handrubbing technique amongst healthcare workers: a controlled, before-after study. PLoS ONE.

[12] Pires D, Bellissimo-Rodrigues F, Soule H, Gayet-Ageron A, Pittet D (2017). Revisiting the WHO "How to Handrub" hand hygiene technique: fingertips first?. Infect Control Hosp Epidemiol.

[13] Boyce JM, Pittet D (2002). Guideline for hand hygiene in health-care settings: recommendations of the healthcare infection control practices advisory committee and the HICPAC/SHEA/APIC/IDSA hand hygiene task force. Infect Control Hosp Epidemiol.

[14] Tschudin-Sutter S, Sepulcri D, Dangel M, Ulrich A, Frei R, Widmer AF (2019). Simplifying the world health organization protocol: 3 steps versus 6 steps for performance of hand hygiene in a cluster-randomized trial. Clin Infect Dis.

[15] Reilly JS, Price L, Lang S, Robertson C, Cheater F, Skinner K, Chow A (2016). A pragmatic randomized controlled trial of 6-step vs 3-step hand hygiene technique in acute hospital care in the United Kingdom. Infect Control Hosp Epidemiol.

[16] Price L, Gozdzielewska L, Matuluko A, Pittet D, Allegranzi B, Reilly J (2022). Comparing the effectiveness of hand hygiene techniques in reducing the microbial load and covering hand surfaces in healthcare workers: Updated systematic review. Am J Infect Control.

[17] Boyce JM (2021). Hand hygiene, an update. Infect Dis Clin North Am.

[18] Leslie R, Donskey C, Zabarsky T, Parker A, Macinga D, Assadian O (2015). Measuring alcohol-based hand rub volume used by healthcare workers in practice. Antimicrobial Resis Infect Control..

[19] Kenters N, Eikelenboom-Boskamp A, Hines J, McGeer A, Huijskens EGW, Voss A (2020). Product dose considerations for real-world hand sanitiser efficacy. Am J Infect Control.

[20] Widmer AE, Dangel M (2004). Alcohol-based handrub: evaluation of technique and microbiological efficacy with international infection control professionals. Infect Control Hosp Epidemiol.

[21] Wilkinson MAC, Ormandy K, Bradley CR, Hines J (2018). Comparison of the efficacy and drying times of liquid, gel and foam formats of alcohol-based hand rubs. J Hosp Infect.

[22] Macinga DR, Shumaker DJ, Werner HP, Edmonds SL, Leslie RA, Parker AE, Arbogast JW (2014). The relative influences of product volume, delivery format and alcohol concentration on dry-time and efficacy of alcohol-based hand rubs. BMC Infect Dis.

[23] Suchomel M, Leslie RA, Parker AE, Macinga DR (2018). How long is enough? Identification of product dry-time as a primary driver of alcohol-based hand rub efficacy. Antimicrob Resist Infect Control.

[24] Chemical disinfectants and antiseptics Hygienic handrub. Test method and requirements (phase 2/step 2), EN 1500 :1997 (1997).

[25] Martinello RA, Arbogast JW, Guercia K, Parker AE, Boyce JM (2019). Nursing preference for alcohol-based hand rub volume. Infect Control Hosp Epidemiol.

[26] Bellissimo-Rodrigues F, Soule H, Gayet-Ageron A, Martin Y, Pittet D (2016). Should alcohol-based handrub use be customized to healthcare workers' hand size?. Infect Control Hosp Epidemiol.

[27] Vermeil T, Peters A, Kilpatrick C, Pires D, Allegranzi B, Pittet D (2019). Hand hygiene in hospitals: anatomy of a revolution. J Hosp Infect.

[28] Pires D, Soule H, Bellissimo-Rodrigues F, Gayet-Ageron A, Pittet D (2017). Hand hygiene with alcohol-based hand rub: how long is long enough?. Infect Control Hosp Epidemiol.

[29] Paula H, Becker R, Assadian O, Heidecke CD, Kramer A (2018). Wettability of hands during 15-second and 30-second handrub time intervals: a prospective, randomized crossover study. Am J Infect Control.

[30] Kramer A, Pittet D, Klasinc R, Krebs S, Koburger T, Fusch C, Assadian O (2017). Shortening the application time of alcohol-based hand rubs to 15 seconds may improve the frequency of hand antisepsis actions in a neonatal intensive care unit. Infect Control Hosp Epidemiol.

[31] Harnoss JC, Dancer SJ, Kaden CF, Baguhl R, Kohlmann T, Papke R, Zygmunt M, Assadian O, Suchomel M (2020). Hand antisepsis without decreasing efficacy by shortening the rub-in time of alcohol-based handrubs to 15 seconds. J Hosp Infect.

[32] Park HY, Kim SK, Lim YJ, Kwak SH, Hong MJ, Mun HM, Park SY, Kim HJ, Choi HR (2014). Assessment of the appropriateness of hand surface coverage for health care workers according to World Health Organization hand hygiene guidelines. Am J Infect Control.

[33] Glowicz JB, Landon E, Sickbert-Bennett EE, Aiello AE, deKay K, Hoffmann KK, Maragakis L, Olmsted RN, Polgreen PM (2023). SHEA/IDSA/APIC practice recommendation: strategies to prevent healthcare-associated infections through hand hygiene: 2022 update. Infect Control Hosp Epidemiol.

[34] Price L, Gozdzielewska L, Alejandre JC, Jorgenson A, Stewart E, Pittet D, Reilly J (2022). Systematic review on factors influencing the effectiveness of alcohol-based hand rubbing in healthcare. Antimicrob Resist Infect Control.

[35] Edmonds SL, Macinga DR, Mays-Suko P, Duley C, Rutter J, Jarvis WR, Arbogast JW (2012). Comparative efficacy of commercially available alcohol-based hand rubs and World Health Organization-recommended hand rubs: formulation matters. Am J Infect Control.

[36] Barbut F, Maury E, Goldwirt L, Boëlle PY, Neyme D, Aman R, Rossi B, Offenstadt G (2007). Comparison of the antibacterial efficacy and acceptability of an alcohol-based hand rinse with two alcohol-based hand gels during routine patient care. J Hosp Infect.

[37] Verwilghen D, Osiak K, Shaw AD, Averay K, Kampf G, van Galen G (2021). Identifying drivers for user preference and acceptability of different hydro-alcoholic hand rub formulations. J Hosp Infect.

[38] Traore O, Hugonnet S, Lübbe J, Griffiths W, Pittet D (2007). Liquid versus gel handrub formulation: a prospective intervention study. Crit Care.

[39] Pittet D, Hugonnet S, Harbarth S, Mourouga P, Sauvan V, Touveneau S, Perneger TV (2000). Effectiveness of a hospital-wide programme to improve compliance with hand hygiene. Infection Control Programme Lancet.

[40] Peters A, Cave C, Carry J, Sauser J, Pittet D (2022). Tolerability and acceptability of three alcohol-based hand-rub gel formulations: a randomized crossover study. J Hosp Infect.

[41] EN1500:2017–10. Chemical disinfectants and antiseptics. Hygienic hand disinfection. Test method and requirement (Phase 2, Step 2). Comité Européen de Normalisation, Brussels; 2017.

[42] ASTM-E2755–10. Standard test method for determining the bacteria-eliminating effectiveness of hand sanitizer formulations using hands of adults; 2010.

[43] Suchomel M, Brill FHH, Kampf G, Leslie RA, Macinga DR (2023). Evolving the EN 1500 test method for alcohol-based hand rub closer to clinical reality by reducing the organic load on hands and enabling product to be applied to dry hands. J Hosp Infect.

[44] Edmonds-Wilson SL, Nurinova NI, Zapka CA, Fierer N, Wilson M (2015). Review of human hand microbiome research. J Dermatol Sci.

[45] Axel K, Mathilde Borg Dahl, Roald Papke, Hortense Slevogt, Haitao Wang, Paula Zwicker, Matthias Heckmann, Anne Reinhard, Mareike Meister, et al. Stabilization of the hand microbiome by alcohol‐based hand antisepsis. Antimicrob Resist Infect Contr. 2023;12:040.

[46] Bauer-Savage J, Pittet D, Kim E, Allegranzi B (2013). Local production of WHO-recommended alcohol-based handrubs: feasibility, advantages, barriers and costs. Bull World Health Organ.

[47] Sax H, Allegranzi B, Uçkay I, Larson E, Boyce J, Pittet D (2007). 'My five moments for hand hygiene': a user-centred design approach to understand, train, monitor and report hand hygiene. J Hosp Infect.

[48] Jeanes A, Coen PG, Gould DJ, Drey NS (2019). Validity of hand hygiene compliance measurement by observation: a systematic review. Am J Infect Control.

[49] Chen LF, Vander Weg MW, Hofmann DA, Reisinger HS (2015). The hawthorne effect in infection prevention and epidemiology. Infect Control Hosp Epidemiol.

[50] Purssell E, Drey N, Chudleigh J, Creedon S, Gould DJ (2020). The Hawthorne effect on adherence to hand hygiene in patient care. J Hosp Infect.

[51] Hagel S, Reischke J, Kesselmeier M, Winning J, Gastmeier P, Brunkhorst FM, Scherag A, Pletz MW (2015). Quantifying the hawthorne effect in hand hygiene compliance through comparing direct observation with automated hand hygiene monitoring. Infect Control Hosp Epidemiol.

[52] Boyce JM (2019). Current issues in hand hygiene. Am J Infect Control.

[53] Gould D, Lindström H, Purssell E, Wigglesworth N (2020). Electronic hand hygiene monitoring: accuracy, impact on the Hawthorne effect and efficiency. J Infect Prev.

[54] Lotfinejad N, Peters A, Tartari E, Fankhauser-Rodriguez C, Pires D, Pittet D (2021). Hand hygiene in health care: 20 years of ongoing advances and perspectives. Lancet Infect Dis.

[55] Hansen MB (2022). Validation of electronic hand hygiene monitoring systems: the IPC community must agree on four essentials. J Hosp Infect.

[56] Limper HM, Slawsky L, Garcia-Houchins S, Mehta S, Hershow RC, Landon E (2017). Assessment of an aggregate-level hand hygiene monitoring technology for measuring hand hygiene performance among healthcare personnel. Infect Control Hosp Epidemiol.

[57] Zwicker P, Meng M, Friesecke S, Stein T, Herzog A, Herzer C, Kammerlander M, Gebhardt T, Kugler C (2023). An interactive feedback system for increasing hand antisepsis adherence in stationary intensive care. J Hosp Infect.

[58] Cao J, Min L, Lansing B, Foxman B, Mody L (2016). Multidrug-resistant organisms on patients' hands: a missed opportunity. JAMA Intern Med.

[59] Dunn AN, Donskey CJ, Gordon SM, Deshpande A (2020). Multidrug-resistant organisms on patients hands in an ICU setting. Infect Control Hosp Epidemiol.

[60] O'Donnell M, Harris T, Horn T, Midamba B, Primes V, Sullivan N, Shuler R, Zabarsky TF, Deshpande A (2015). Sustained increase in resident meal time hand hygiene through an interdisciplinary intervention engaging long-term care facility residents and staff. Am J Infect Control.

[61] Loveday HP, Tingle A, Wilson JA (2021). Using a multimodal strategy to improve patient hand hygiene. Am J Infect Control.

[62] Sunkesula VCK, Kundrapu S, Knighton S, Cadnum JL, Donskey CJ (2017). A randomized trial to determine the impact of an educational patient hand-hygiene intervention on contamination of hospitalized patient's hands with healthcare-associated pathogens. Infect Control Hosp Epidemiol.

[63] Cheng VC, Wu AK, Cheung CH, Lau SK, Woo PC, Chan KH, Li KS, Ip IK, Dunn EL (2007). Outbreak of human metapneumovirus infection in psychiatric inpatients: implications for directly observed use of alcohol hand rub in prevention of nosocomial outbreaks. J Hosp Infect.

[64] Gagné D, Bédard G, Maziade PJ (2010). Systematic patients' hand disinfection: impact on meticillin-resistant Staphylococcus aureus infection rates in a community hospital. J Hosp Infect.

[65] Cheng VC, Tai JW, Chau PH, Lai CK, Chuang VW, So SY, Wong SC, Chen JH, Ho PL (2016). Successful control of emerging vancomycin-resistant enterococci by territory-wide implementation of directly observed hand hygiene in patients in Hong Kong. Am J Infect Control.

[66] Pokrywka M, Buraczewski M, Frank D, Dixon H, Ferrelli J, Shutt K, Yassin M (2017). Can improving patient hand hygiene impact Clostridium difficile infection events at an academic medical center?. Am J Infect Control.

[67] Rai H, Saldana C, Gonzalez-Orta MI, Knighton S, Cadnum JL, Donskey CJ (2019). A pilot study to assess the impact of an educational patient hand hygiene intervention on acquisition of colonization with health care-associated pathogens. Am J Infect Control.

[68] WHO. Global report on infection prevention and control. Geneva, Switzerland; 2022. Report No.: CC BY-NC-SA 3.0 IGO.

[69] WHO. Global strategy on infection prevention and control. Geneva, Switzerland; 2023.

[70] World Health Organization (2023). My 5 moments for hand hygiene.

[71] World Health Organization (2023). WHO research for hand hygiene in health care 2023–2030: summary.

